# 

**Published:** 1884-02-20

**Authors:** 


*The Doctors of New Orleans* are rejoicing over a recent decision of the court touching the fees of the physicians appointed to examine vessels at quarantine. The owners of vessels filed an injunction, refusing to pay the fees, and the matter was adjudicated, resulting in the success of the Doctors.

*The American Public Health Association*— Will meet on November 13th, at Detroit, Michigan, and continue in session several days, during which papers will be read on some very important subjects. A full attendance is expected.

*The New York Post Graduate Medical School*—Has been so successful that on or about February 1st it moved to a new building, which enables it to give hospital advantages to its matriculates. The new building is very large, being five stories high and having a front of 95 feet. The new announcement gives a list of 140 physicians who were matriculates for the year ending November 1, 1883.

### *RECEIPTED.*

1884.—Drs A Atkinson, P C Sircuit, E A Cox, J W Baker, J C Wallis, R L Hinton, S M Hogan, V T Lindsay, Alison Lockwood, S C Spearman, P Taylor, L S Brownlee, Eugene Fowler, James Jordan, Eli Smythe, R S Tomaston, Edward Burk, M M Evans, J B Barwick, E A Anderson to July '85; J S Bushong, '82; J L Dunent, '83; J McCullom, '83; R M Canuth, to Oct '83; T L Turk, '84.

